# Health-related quality of life using specific and generic questionnaires in Spanish coeliac children

**DOI:** 10.1186/s12955-020-01494-x

**Published:** 2020-07-25

**Authors:** Josefa Barrio, Maria Luz Cilleruelo, Enriqueta Román, Cristina Fernández

**Affiliations:** 1 Department of Paediatrics, Hospital Universitario de Fuenlabrada, Madrid, Spain; 2 Department of Paediatrics, Hospital Universitario Puerta Hierro, Madrid, Spain; 3 Department of Epidemiology, Hospital Universitario Clínico de Madrid, Madrid, Spain

**Keywords:** Coeliac disease, Quality of life, Questionnaires, Correlation, Health outcomes

## Abstract

**Background:**

We aimed to compare the perception of health-related quality of life (HRQOL) and related factors in Spanish coeliac children and their parents, using two questionnaires, the generic KIDSCREEN-52 and the specific the Celiac Disease DUX (CDDUX), and to assess the correlation between them.

**Methods:**

Coeliac children, aged 8-18, who are members of the Madrid Coeliac Association (MCA) and their parents, answered the Spanish version of the CDDUX and KIDSCREEN-52 questionnaires via e-mail. CDDUX was answered by 266 children and 428 parents and KIDSCREEN-52 by 255 children and 387 parents. Linear regression models were fitted to evaluate the association of demographic and clinical factors with HRQOL scores. CDDUX scores were compared with the subjective perception of health status assessed by the first question of KIDSCREEN-52. The correlation between the questionnaires was analysed.

**Results:**

We found that the main factors that negatively affected HRQOL were having social or economic difficulties associated with following the diet and having transgression-related symptoms. The maximum correlation between the questionnaires was 0.309 and − 0.254 in parents and children respectively.

**Conclusions:**

Although there is a poor correlation between the two questionnaires, both agreed that the main concerns of the respondents were related to the social and economic difficulties of following the diet. It would be interesting to use both types of questionnaires in order to perform a more complete assessment of HRQOL in coeliac children.

**Trial registration:**

Not applicable.

## Introduction

Coeliac disease (CD) is an immune-mediated, systemic disorder due to gluten ingestion in genetically susceptible individuals [1]. Gluten-free diet (GFD) is currently the only effective treatment for CD. Although adherence to the GFD resolves intestinal lesion and related symptoms, the obligation to follow a strict, restrictive and permanent diet, together with the chronic nature of this illness, can have a considerable negative impact on coeliac patients and their families’ health-related quality of life (HRQOL) [2, 3] For these reasons, it is interesting to evaluate HRQOL in CD children and their parents and how the disease impacts on their lives. Over the last few years, the growing interest in evaluating HRQOL in CD children has led to the publication of many studies [4–7]. However, in Spain, it has not been studied sufficiently. For this reason, it was necessary to study it.

The best way to evaluate HRQOL is using questionnaires. There are two main types, the generic and specific questionnaires. Generic questionnaires measure the daily life aspects of HRQOL in patients who have several conditions, whereas specific questionnaires focus on specific aspects related to the illness and its treatment [8].

The two questionnaires chosen to perform our study were the KIDSCREEN-52 and the CDDUX. The KIDSCREEN-52 questionnaire was chosen because it is one of the most widely used generic questionnaires worldwide [9] and has been translated into Spanish [10]. The Coeliac Disease DUX (CDDUX) [11] was chosen because it is the first paediatric disease-specific HRQOL questionnaire, it was recently adapted into Spanish by our group [12] and is also available in other languages [13–15].

Most HRQOL studies performed with coeliac children using either specific [13, 14, 16] or generic questionnaires [16–18] have observed no substantial negative impact on parents’ and children’s HRQOL, which is similar to our findings using the CDDUX [12] and the KIDSCREEN-52 [19]. However, some authors have found some negative impact [11, 15]. Not many studies have evaluated coeliac children’s HRQOL using a generic and a specific questionnaire. It is interesting to use both types of questionnaires because the information obtained with them is different, it can be complementary and useful for better assessing HRQOL.

The purpose of this study was to compare HRQOL outcomes and related factors (demographics: age, gender, parent educational level, living conditions as well as clinical factors: age upon diagnosis, time since diagnosis, clinical presentation at onset, family history of CD, associated diseases and disease follow-up data such as adherence to treatment, difficulties in following the diet and whether symptoms reappeared when gluten was ingested) in a group of coeliac children and their parents by using both the generic KIDSCREEN-52 and the specific CDDUX questionnaires, which focus on different aspects of the disease. A second objective was to assess whether there is a correlation between the results obtained with both questionnaires.

## Materials and methods

### Ethics

The study protocol was approved by the Clinical Research Ethics Committee of the Hospital Universitario de Fuenlabrada, Madrid, Spain. Informed consent was obtained from all parents or guardians and from all patients aged 12 years or older.

### Study participants

This is a cross-sectional survey targeted at children from Madrid with CD aged 8 to 18 and their parents [12, 19]. In order to get a good representation of patients from around the Autonomous Region of Madrid, we contacted the Madrid Coeliac Association (MCA). Potential candidates were identified among their members in the target age range. Information about the study was sent via e-mail so as to reach the largest number of patients. Information about the study was sent to 1602 coeliac children, aged 8 to 18, with an e-mail address associated with the MCA at that moment. Children and parents were instructed to independently access a link from the MCA webpage containing instructions and information about the study. The information sent included an informed consent form, the Spanish versions of the two questionnaires, demographic and clinical data. The CDDUX was the first questionnaire which people had access to, in order to prioritize answering the specific questionnaire, and, subsequently, they could access the KIDSCREEN-52 questionnaire.

The sample size was calculated according to the variance shown by the items in the questionnaire validation study [11]. The sample size required for 3% accuracy and a 95% level of confidence (95% CI) was 222 children and their parents. The recruitment process was halted upon acquiring the necessary number of participants.

As previously published elsewhere [12, 19], our sample was representative of the MCA population in terms of age and gender.

Demographic data recorded were: age, gender, parent educational level and living conditions. Clinical data collected were: age upon diagnosis, time since diagnosis, clinical presentation at onset, family history of CD, associated diseases and disease follow-up data such as adherence to treatment, difficulties in following the diet and whether symptoms reappeared when gluten was ingested.

Participants were stratified by age upon questionnaire completion in: children (8-11 years old), young adolescents (12-15 years old) or older adolescents (16-18 years old), by age upon CD diagnosis (< 2–7 and >  7 years old), and by years since diagnosis (< 4, 4-8 and > 8 years old).

Factors related to QOL (demographic and clinical data) were analysed according to the total number of answers provided by the 428 patients whose parents answered the CDDUX questionnaire and by 387 patients whose parents answered to the KIDSCREEN-52 questionnaire (41 parents only answered the CDDUX questionnaire).

For the comparisons between both questionnaires, only the 387 and 255 parents and children’s matched CDDUX and KIDSCREEN-52 questionnaires were taken into account.

The findings obtained in the univariate analysis using both questionnaires have been reported elsewhere [12, 19]. This present study provides results obtained in the multivariate analysis, as well as the comparison and correlation between the questionnaires.

### Questionnaire descriptions

Two questionnaires were used: the Spanish versions of the specific CDDUX Coeliac disease questionnaire and the generic KIDSCREEN-52 questionnaire.

The Spanish version of the CDDUX questionnaire, which was previously validated by our group [12], contains 12 items distributed in 3 scales: “having CD” (3 items), which provides information on how the child feels when offered food that contains gluten or when thinking about food containing gluten; “communication” (3 items), which provides information about how the child feels when talking about CD to others or when explaining what the disease is; and “diet” (6 items), which provides information on how the child feels about having to follow a strict, lifelong diet or not being able to eat things that other people eat. Each item has 5 response options, aided by a picture diagram with faces expressing different corresponding emotional states**.** The CDDUX scores were recoded into a scale from 1 to 100, with 1 being very bad and 100 very good. When using a 5-point Likert scale, a score of 1–20 is considered very bad, 21–40 is bad, 41–60 is neutral, 61–80 is good, and 81 to 100 is very good.

The Spanish version of the generic KIDSCREEN-52 questionnaire consists of 52 items covering 10 QOL domains [9, 10]: “social acceptance” (bullying), “moods and emotions”, “physical well-being”, “psychological well-being”, “self-perception”, “school environment”, “parent relations and family life”, “economic resources”, “autonomy” and “social support and peers”. Moreover, in the questionnaire’s first question, the child or parent is asked about how they perceive their own, or their child’s, state of health (excellent, very good, good, not bad/not good, bad). The responses to the other 51 questions marked by patients and parents were transformed into a 5-point Likert-type scale in order to assess either the frequency (never, seldom, sometimes, often, always) of certain behaviours/feelings or the intensity of an attitude (not at all, slightly moderately, very, extremely). Children and adolescents were asked to select one response by recalling their situation over the period of the previous week by themselves. HRQOL was recorded on a scale from 1 to 100, with higher scores meaning a better HRQOL, like in the original version. Like with the CDDUX, HRQOL was considered “very bad” for scores 1–20, “bad” for scores 21-40, “neutral” for scores 41–60, “good” for scores 61–80 or “very good” for scores 81–100. Both questionnaires have a version for children/adolescents aged 8 to 18 and another one for parents which had to be independently completed.

### Statistical analysis

Kolmogorov-Smirnov (K-S) test was used to analyse the normality of variables. Results referring to the questionnaire scores in the different dimensions were provided as a mean (SD). Mean scores were compared by ANOVA according to the clinical and demographic variables included. Linear regression models were fitted to evaluate the association of the independent variables (demographic and clinical) with the dependent variables (HRQOL scores). Models were constructed including those variables which obtained a statistical significance in the univariate analysis (*p* < 0.05). CDDUX scores (quantitative variable) were compared with the subjective perception of health status assessed by the first question of KIDSCREEN-52. To evaluate the correlation between the dimensions of both questionnaires, the Pearson correlation coefficient (r) and its statistical test were used.

All statistical tests were performed using the software package SPSS 15.0 for Windows (SPSS Inc., Chicago, IL, USA). Significance was set at *p* < 0.05.

## Results

**Fig. 1:**
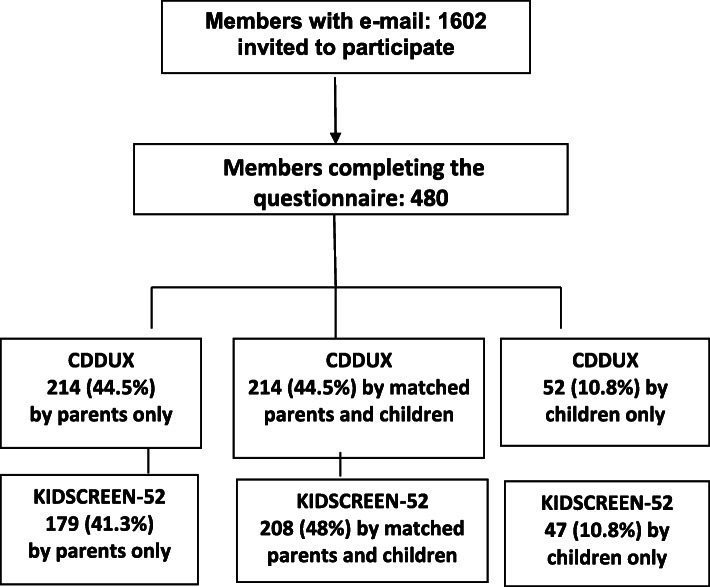
Flowchart with the selection of the population included in the study

The flowchart with the population included in the study is shown in Fig. 1. Demographics and clinical variables, are included in Table 1.

**Table 1:** Demographics and clinical variables

	CDDUX	KIDSCREEN-52
**CHILDREN (replies children/parents)**	480 (266/214)	434 (255/179)
Girls	288 (60%)	269 (62%)
**Age (years)**	N (%)	N (%)
8-11	188 (39. 2%)	167 (38. 5%)
12-15	225 (46.9%)	206 (47. 5%)
16-18	67 (14%)	61 (14. 1%)
**Mean age (years)**	12.4 (SD 2.8)	12.4 (SD 2.8)
**Median age at diagnosis (years)**	2 (IQR 2–5)	2 (IQR2–5)
**<** 2	60%	61.3%
3-**7**	22%	20,3%
**> 7**	18%	18.4%
**Mean time since diagnosis (years)**	8.4 (SD 4. 2)	8.4 (SD 4. 2)
**< 4**	18. 6%	17.3%
5**–8**	33.4%	32.9%
**> 7**	48%	49.8%
**Family history of CD**	20. 6%	20.7%
**Disease manifestation at onset**		
Classic form	263 (61.4%)	239 (61.8%)
Non classic form	160 (37.4%)	143 (36.9%)
Dermatitis herpetiformis	2 (0. 5%)	2 (0. 5%)
During screening	3 (0.7%)	3 (0.7%)
**Associated conditions**	34 (12. 6%)	34 (12. 6%)
**Medical follow-up**		
• Every two years	12. 1%	10.9%
• Once a year	71. 5%	71. 5%
• Every six months	11.4%	13.4%
• Every three months	1. 6%	1.7%
• Occasionally	1. 2%	1.3%
• Never	2. 2%	1.3%
**Adherence to the diet**		
• Always 96. 6%	414(96.7%)	374(96. 6%)
• Most times 3. 1%	13 (3.03%)	12(3. 1%)
• Sometimes 0.3%	1(0.3%)	1(0.3%)
**Difficulties with the diet**		
• No	380 (88.7%)	346(89.4%)
• Economic	4(0.93%)	4(1%)
• Social	12 (2.8%)	6(11%)
• Eating out	32 (7.4%)	26(6.7%)

*SD* Standard deviation, *IQR* Interquartile range, *CD* Coeliac Disease

Classic form: signs and symptoms of malabsorption. Diarrhoea, steatorrhea, weight loss or growth failure is required. Oslo 2012

Non-classic form: presents without signs and symptoms of malabsorption. (Oslo 2012)

Associated conditions: Diseases with increased prevalence of CD

### HRQOL

Mean total (SD) HRQOL CDDUX scores were 55. 5 (SD 12.7) and 53.89 (SD 12.19) in children and parents respectively, with no differences detected in paired comparisons between the two groups, except in the dimension “having coeliac disease”, in which parents scored significantly higher than children (*p* < 0.001). (Table 1 of supplemental digital content). Whereas mean KIDSCREEN-52 scores ranged from 91.92 (SD 11.91) in the dimension “social acceptance” to 32.18 (SD 11.82) in “social support and peers” children and parents scored over 40 in 8 out of 10 KIDSCREEN-52 dimensions. Nevertheless, both scored their HRQOL as “bad” (scores between 20 and 40) in two dimensions: “autonomy” and “social support and peers.” Children scored higher than parents in 5 dimensions while parents rated “social support and peers” higher. (*p* < 0.001). (Table 2 of supplemental digital content).

### QOL-related factors

According to the fitted lineal regression models, non-classical presentation at onset (− 3.71, 95% CI -6.44, − 0.98, *p* = 0.008) and having economic or social difficulties with adhering to the diet (− 6.69, 95% CI -10.59, − 2.78, *p* = 0.001) were detected as factors related to a decrease in HRQOL scores in the total mean CDDUX scores. Children having symptoms with transgressions (− 2.70; IC 95% -5.16; − 0.25; *p* = 0.03) and those with less than 4 years since diagnosis (− 4.70; IC 95%-7.91; − 1.48; *p* = 0.004) scored lower in the “having CD” dimension. In addition, children younger than 2 years upon diagnosis scored better (3.8; IC 95% 0.8; 6.8; *p* < 0.001) only on the communication scale. Patients with adherence to treatment scored higher in HRQOL in the total score (6.04, 95% CI -0.62, 12.70, *p* = 0.075) and in the communication and diet dimensions (Table 2)

**Table 2:** HRQOL factors using CDDUX, based on scores given by the 428 parents who answered the questionnaire (linear regression models)

		Beta^a^	***p-***value	95% CI	
**TOTAL SCORE**	**Non-Classical form vs others**	**−3.71**	**0.008**	**-6.44**	**− 0.98**
	**Adherence to diet Yes/No**	6**.04**	**0.075**	**− 0.62**	**12.70**
	**Economic or social difficulties** **vs. others**	**-**6**.69**	**0.001**	**−10.60**	**-**2**.78**
**HAVING CD**	**Time since diagnosis (years)**				
	**< 4 vs. > 8**	**− 4.69**	**0.004**	**−7.91**	**-**1**.48**
	4-**8 vs. > 8**	**− 3.80**	**0.005**	**-**6**.43**	**-**1**.16**
	**Economic or social difficulties vs. others**	**−4.55**	**0.022**	**− 8,.44**	**− 0.67**
	**Transgression symptoms yes/ no**	**-**2**.70**	**0.031**	**-**5**.16**	**−0.25**
	**University studies: mother/ rest**	**4.39**	**< 0.001**	2**.14**	6**.63**
**COMMUNICATION**	**Age upon diagnosis (years) (<** 2 **vs. =>** 2**)**	**3.87**	**0.012**	**0.87**	6**.88**
	**Adherence to diet Yes/No**	**10.96**	**0.009**	2**.71**	**19.21**
	**Economic or social difficulties vs. others**	**-**5**.81**	**0.021**	**−10.73**	**− 0.90**
	**University studies: father vs others**	**−3.98**	**0.007**	**-**6**.85**	**-**1**.12**
**DIET**	**Non-Classical form vs. others**	**−4.50**	**0.011**	**−7.99**	**-**1**.02**
	**Adherence to diet (yes / no)**	**8.36**	**0.054**	**−0.13**	**16.87**
	**Economic or social difficulties vs.** **others**	**−8.83**	**0.001**	**−13.82**	**−3.84**

^a^Beta absolute mean effect; *CI* Confidence interval, *CD* Coeliac Disease, *HRQOL* Health-related Quality of life

.

When the KIDSCREEN-52 questionnaire was used (Table 3), some demographic factors were found to be related to a decrease in HRQOL: age older than twelve at the time the questionnaire was filled out (in mood and emotions, psychological and physical wellbeing and school dimensions); female gender (in psychological and physical wellbeing dimensions); and a time since diagnosis of less than 4 years (in psychological wellbeing and self-esteem dimensions). Furthermore, a decrease in HRQOL was observed in children who reported having economic and social difficulties in adhering to the diet and those having symptoms with transgressions.

**Table 3:** HRQOL factors using KIDSCREEN-52, based on scores given by *n* = 387 parents (linear regression models)

		Beta^a^	***p-***value	IC for B at 95%	
**Social Acceptance**	**Social and economic difficulties vs. others**	**-**6**.386**	**0.002**	**−10.31**	**-**2**.46**
**Mood and emotions**	**Age (**12-**15 /** 8-11**)**	**-**2**.82**	**0.024**	**-**5**.28**	**−0.37**
	**Age (**16-**18 /** 8-11**)**	**−4.88**	**0.010**	**−8.56**	**-**1**.19**
	**Social and economic difficulties vs others**	**-**6**.23**	**0.002**	**−10.13**	**-**2**.34**
	**Symptoms with transgressions**	**−3.19**	**0.009**	**-**5**.57**	**−0.80**
**Psychological wellbeing**	**Age (**12-**15 /** 8-11**)**	**-**2**.02**	**0.033**	**−3.87**	**−0.17**
	**Age (**16-**18 /** 8-11**)**	**-**6**.27**	**< 0.001**	**− 9.02**	**− 3.51**
	**Gender (boy /girl)**	2**.87**	**0.002**	1**.10**	**4.65**
	**Time since diagnosis (< 4/=0 > 4)**	**-**1**.93**	**0.031**	**−3.68**	**− 0.18**
	**Mother secondary studies /** 1	**− 3.43**	**0.006**	**-**5**.68**	**-**1**.00**
	**Mother University studies /** 1	**-**1**.99**	**0.015**	**−3.59**	**− 0.38**
**Physical wellbeing**	**Age (**12-**15 /** 8-11**)**	**-**1**.65**	**0.061**	**−3.37**	**0.08**
	**Age (**16-**18 /** 8-11**)**	**-**5**.39**	**< 0.001**	**− 8.00**	**-**2**.83**
	**Gender (boy/girl)**	2**.55**	**0.003**	**0.90**	**4.20**
**School environment**	**Symptoms with transgressions yes/no**	**-**1**.36**	**0.004**	**− 0.44**	**-**2**.29**
	**Age (**12-**15/** 8-11**)**	**-**1**.88**	**< 0.001**	**-**2**.84**	**- 0.92**
	**Age (**16-**18/** 8-11**)**	**−3.55**	**< 0.001**	**−4.97**	**-**2**.13**
**Self- perception**	**Time since diagnosis (< 4/> 8)**	2**.26**	**0.068**	**−0.17**	**4.68**
	**Time since diagnosis (**4-**8 /> 8)**	1**.90**	**0.091**	**−0.30**	**4.10**
	**Age (**12-**15 /** 8-11**)**	**-**2**.07**	**0.050**	**−4.13**	**−0.00**
	**Age (**16-**18 /** 8-11**)**	**−4.58**	**0.002**	**−7.53**	**-**1**.63**
	**Social/economic difficulties vs. other**	**-**5**.03**	**0.001**	**−7.90**	**-**2**.15**

^a^Beta absolute mean effect; *CI* Confidence interval

### Comparison of children’s and parents’ CDDUX scores and the subjective assessment of their health status

We compared mean scores of the children’s (*n* = 255) and parents’ (*n* = 387) CDDUX questionnaires regarding the subjective assessment of their health status (the first question of the KIDSCREEN-52 questionnaire) because the latter, in one single question, provides a general subjective idea about how respondents consider their own health status, so it is interesting to know if there is an agreement or not between the outcomes obtained with both questionnaires.

We observed that the higher the CDDUX scores observed in children and parents, the better the health status assessment (*p* < 0.01) in the total score and diet dimension. However, higher CDDUX scores observed in children and parents did not show a similar improvement in the health status assessment in the “having CD” dimension for children or parents nor in the communications dimension pertaining to the children (Table 4).

**Table 4:** Comparison between the first question in the KIDSCREEN-52, in relation to the subjective perception of health status, and the CDDUX scores (in the *n* = 255 children and *N* = 387 parents answered both questionnaires)

		N	Children CDDUX Mean SCORES (SD)	***p***-value		Parents CDDUX Mean SCORES (SD)	***p-***value
**Total**	**Not bad-not good**	5	**50.67 (12.62)**	**0.01**	**12**	**50.14 (15.69)**	**< 0.001**
	**Good**	**43**	**50.43 (12.72)**		**93**	**50.14 (10.69)**	
	**Very good**	**122**	**55.23 (12.22)**		**162**	**54.87(**11**.83)**	
	**Excellent**	**85**	**58.33 (12.93)**		**120**	**58.88(13.37)**	
	**Total**	**255**	**55.37 (12.78)**		**387**	**54.83(12.60)**	
**Having CD**	**Not bad-not good**	5	**52.00 (**5**.58)**	**0.63**	**12**	**46.** 11**(14.90)**	**0.15**
	**Good**	**43**	**45.12 (12.07)**		**93**	**47.60(12.42)**	
	**Very good**	**122**	**46.99 (12.88)**		**162**	**49.51(12.58)**	
	**Excellent**	**85**	**45.80 (14.28)**		**120**	**51.28(12.81)**	
	**Total**	**255**	**46.38 (13.13)**		**387**	**49.49(12.73)**	
**Communication**	**Not bad-not good**	5	**69.33 (19.78)**	**0.27**	**12**	**61.67(10.68)**	**< 0.001**
	**Good**	**43**	**68.99 (18.91)**		**93**	**64.95(13.72)**	
	**Very good**	**122**	**70.98 (16.42)**		**162**	**69.51(15.64)**	
	**Excellent**	**85**	**74.67 (16.83)**		**120**	**74.78(16.56)**	
	**Total**	**255**	**71.84 (17.09)**		**387**	**69.80(15.82)**	
**Diet**	**Not bad-not good**	5	**40.67 (15.53)**	**< 0.001**	**12**	**46.39(20.96)**	**< 0.001**
	**Good**	**43**	**43.80 (16.91)**		**93**	**44.01(13.96)**	
	**Very good**	**122**	**51.48 (15.79)**		**162**	**50.23(14.60)**	
	**Excellent**	**85**	**56.43 (18.33)**		**120**	**54.72(17.96)**	
	**Total**	**255**	**51.62 (17.36)**		**387**	**50.01(16.23)**	

*SD* Standard deviation, *CD* Coeliac Disease

### Correlation between the questionnaires

The correlation between the questionnaires was poor, being worse in children than parents. The maximum correlation obtained was 0.309 in parents and − 0.254 in children. In children, the maximum correlations were inverse and were obtained between the “autonomy dimension” of KIDSCREEN-52 and all the dimensions of CDDUX. In parents, the best correlations between the questionnaires were positive and were obtained between the “mood and emotions” dimension of KIDSCREEN-52 and the “diet” dimension and total score of CDDUX (Table 5).

**Table 5:** Correlation between CDDUX and KIDSCREEN scores (n = 255 children and n = 387 parents)

		Children	Parents	Children	Parents	Children	Parents	Children	Parents
**Social acceptance**	**Pearson r**	**0.028**	**0.268**	**0.115**	**0.118**	**0.009**	**0.20.243**	**−0.006**	**0.252**
	**p**	0.656	**< 0.001**	0.068	**0.020**	0.891	**< 0.001**	0.920	**< 0.001**
**Moods and emotions**	**Pearson r**	**0.208**	**0.307**	**0.057**	**0.168**	**0.138**	**0.211**	**0.217**	**0.309**
	**p**	0.001	**< 0.001**	0.363	**0.001**	0.028	**< 0.001**	**< 0.001**	**< 0.001**
**Psychological wellbeing**	**Pearson r**	**0.136**	**0.148**	**−0.058**	**0.054**	**0.096**	**0.202**	**0.175**	**0.110**
	**p**	0.030	**0.004**	0.357	**0.292**	0.124	**0.000**	0.005	**0.031**
**Physical wellbeing**	**Pearson r**	**0.159**	**0.181**	**−0.061**	**0.072**	**0.112**	**0.230**	**0.203**	**0.142**
	**p**	0.011	**0.000**	0.328	**0.160**	0.075	**0.000**	0.001	**0.005**
**Self- perception**	**Pearson r**	**0.113**	**0.126**	**−0.064**	**0.013**	**−0.019**	**0.103**	**0.200**	**0.140**
	**p**	0.071	**0.013**	0.309	**0.800**	0.758	**0.042**	0.001	**0.006**
**School environment**	**Pearson r**	**0.012**	**−0.039**	**−0.020**	**−0.080**	**0.028**	**0.031**	**0.011**	**−0.044**
	**p**	0.849	**0.443**	0.753	**0.116**	0.659	**0.548**	0.856	**0.384**
**Parent relations**	**Pearson r**	**−0.064**	**−0.008**	**0.052**	**0.031**	**−0.146**	**−0.022**	**− 0.042**	**−0.014**
	**p**	0.312	**0.875**	0.411	**0.542**	0.020	**0.671**	0.509	**0.783**
**Financial resources**	**Pearson r**	**−0.099**	**−0.051**	**−0.021**	**− 0.006**	**−0.052**	**0.033**	**−0.112**	**− 0.092**
	**p**	0.115	**0.321**	0.733	**0.906**	0.405	**0.518**	0.076	**0.070**
**Autonomy**	**Pearson r**	**−0.253**	**−0.225**	**−0.104**	**− 0.134**	**−0.254**	**− 0.184**	**−0.208**	**−** 0.208
	**p**	0.000	**0.000**	0.096	**0.008**	0.000	**0.000**	0.001	**0.000**
**Social support and peers**	**Pearson r**	**−0.203**	**−0.226**	**− 0.136**	**−0.175**	**− 0.198**	**− 0.190**	**−0.151**	**− 0.189**
	**p**	0.001	**0.000**	0.029	**0.001**	0.002	**0.000**	0.016	**0.000**

## Discussion

In previous studies [12, 19], our group assessed the subjective HRQOL in coeliac children on a GFD and their parents, using two questionnaires, a specific one (CDDUX) and a generic one (KIDSCREEN-52). Coeliac children and their parents showed a neutral HRQOL experience using the CDDUX and a neutral to good HRQOL experience using the KIDSCREEN-52. Children scored higher than parents in most of the KIDSCREEN-52 dimensions, while they scored higher than parents in only one of the CDDUX dimensions. (supplemental digital content Tables 1 and 2). In general, we observed higher scores with the generic questionnaire when compared with the specific one, in line with findings from other investigators [11, 13, 16, 20]. However, in our previous studies, both questionnaires showed some concerns regarding the HRQOL of our patients, which led us to take a deeper look and try to further elucidate factors related to having a worse perception of HRQOL [12, 19].

According to the multivariate analysis of our current study, we observed that the main factors related to having a worse HRQOL with both questionnaires were: having social and/or economic difficulties related to following the diet and having transgression-related symptoms. Moreover, in the CDDUX questionnaire, the non-complete adherence to diet, the non-classical form of CD at diagnosis and being older than 2 years of age upon diagnosis were found to be associated with having worse HRQOL while with the KIDSCREEN-52 questionnaire being female and being over 12 years of age when the survey was filled out were associated with a worse perception of HRQOL. In our previous studies [12, 19] with the CDDUX questionnaire, the univariate analysis showed the same factors related to having a worse HRQOL and these factors have now been confirmed by the multivariate analysis. Regarding the KIDSCREEN-52 questionnaire, non-complete dietary adherence was the only factor related to a worse HRQOL according to the univariate analysis that was not confirmed by the results in the current study. A negative perception of HRQOL in patients reporting non-adherence to the diet and with social or economic difficulties related to following the diet has been reported by other authors in children [13, 15, 21, 22] and adults [23–28] with CD. Feeling different in social and school settings, particularly among adolescents, has been assessed as an important factor influencing HRQOL in coeliac children [29]. Likewise, a negative association between HRQOL and having transgression-related symptoms has been reported by other authors [28].

The CDDUX questionnaire seems to be more sensitive for detecting differences in clinical variables. Therefore, children younger than 2 at the time of diagnosis scored better on their HRQOL, which is in line with reports from other authors [17, 30]. The explanation behind this could be that the younger the child is upon diagnosis, the less accustomed to the taste of gluten-containing food he/she is and, therefore, the better the compliance with the gluten-free diet is. In our current study, the non-classical clinical presentation form for CD was associated with a worse perception of HRQOL according to the CDDUX results. However, no differences were found in clinical presentation with the KIDSCREEN-52 questionnaire, nor did other authors who used different questionnaires, such as Bystrom [17] and Choung [31], find them. In spite of this, the KIDSCREEN-52 questionnaire detected more differences in demographic variables, such as gender and age at the time of filling out the survey, as reflected in worse scores in children older than 12 years of age and in females. Nevertheless, neither our group using the Spanish CDDUX, nor other investigators that used the same questionnaire found differences in relation to those factors [11, 13, 17]. In both questionnaires, the group of children with less time since diagnosis experienced some aspects of their HRQOL as lower. The explanation could be related to a better acceptance of the disease and the diet as the time from diagnosis increases.

Mean CDDUX scores were compared with the subjective assessment of health status, as assessed with the first question of KIDSCREEN-52. Results showed that when patients had a better perception of their health status, the mean CDDUX scores were better as well, which agrees with the results obtained by other researchers [11].

Regarding our second objective, the correlation between the mean scores of the generic KIDSCREEN-52 and the specific CDDUX questionnaires was also assessed. We observed a poor correlation between the questionnaires, with worse results in children than in parents. These results are in line with those reported by Jordan et al. [16], which observed a poor to moderate correlation between the generic Pediatric Quality of Life Inventory (PedsQL) and the specific CDPQOL questionnaires in coeliac children, and those by Picó et al. [13], which found a moderate correlation between the CDDUX and the generic PedsQL questionnaires. The discrepancies between specific and generic questionnaires can be explained by the different types of information obtained, in addition to the different methodologies used in both of them. Specific CD questionnaires focus on aspects of life that are influenced by having the disease and having to adhere to a restrictive diet throughout life, whereas generic questionnaires assess the different dimensions that condition the general HRQOL. The justification of that can be that the individual may have a good overall HRQOL but have problems related to their disease which are not detected by a generic questionnaire, hence the importance of using both questionnaires [26].

Our study has some limitations. Participants were all members of a coeliac association, which could introduce biases since these families would likely be highly motivated to deal with their child’s problem. Similarly, as an inclusion criterion for this study was having an e-mail address, participants may not have been representative of low-income families. However, our study population is the best possible representation of the whole child CD population in Madrid.

## Conclusions

Although there is a poor correlation between the two questionnaires, both agreed that the main concerns of the respondents were related to the social and economic difficulties of following the diet. It would be interesting to use of both types of questionnaires in order to perform a more complete assessment of HRQOL in coeliac children.

## Supplementary information

**Additional file 1.** Supplemental digital content. Table 1. Health-related quality of life (HRQOL) scores provided by 266 Spanish children with coeliac disease and parents (428) of children with coeliac disease using the specific CDDUX questionnaire. Supplemental digital content. Table 2. Health-related quality of life (HRQOL) scores provided by Spanish children with coeliac disease and parents of children with coeliac disease using the generic KIDSCREEN-52 questionnaire.

## Data Availability

The dataset used for this research will be available from the corresponding author upon reasonable request.

## Abbreviations

CD: Coeliac disease
GFD: Gluten-free diet
MCA: Madrid Coeliac Association
CI: Confidence interval
HRQOL: Health-related quality of life
PedsQL: Pediatric Quality of Life Inventory (PedsQL)
QOL: Quality of life
