# Correction: Health-related quality of life using specific and generic questionnaires in Spanish coeliac children

**DOI:** 10.1186/s12955-023-02138-6

**Published:** 2023-06-07

**Authors:** Josefa Barrio, Maria Luz Cilleruelo, Enriqueta Román, Cristina Fernández

**Affiliations:** 1 Department of Paediatrics, Hospital Universitario de Fuenlabrada, Madrid, Spain; 2 Department of Paediatrics, Hospital Universitario Puerta Hierro, Madrid, Spain; 3 Department of Epidemiology, Hospital Universitario Clínico de Madrid, Madrid, Spain

Health and Quality of Life Outcomes (2020) 18:250

10.1186/s12955-020-01494-x

The original article [1] mistakenly contains incorrect information in the Funding section. The correct Funding statement is as such:

## Funding

This study has been funded by Instituto de Salud Carlos III through the project “PI16/00358” (Co-funded by European Regional Development Fund; “A way to make Europe”).
