# Supplementary material for: Health-related quality of life using specific and generic questionnaires in Spanish coeliac children
Source: Health Qual Life Outcomes. 2020 Jul 25;18:250. doi: 10.1186/s12955-020-01494-x (PMC7382098; doi:10.1186/s12955-020-01494-x)
Supplement: Supplementary file 1 — Additional file 1. Supplemental digital content. Table 1. Health-related quality of life (HRQOL) scores provided by 266 Spanish children with coeliac disease and parents (428) of children with coeliac disease using the specific CDDUX questionnaire. Supplemental digital content. Table 2. Health-related quality of life (HRQOL) scores provided by Spanish children with coeliac disease and parents of children with coeliac disease using the generic KIDSCREEN-52 questionnaire. [file 12955_2020_1494_MOESM1_ESM.doc]

**Supplemental digital content. Table 1.**

**Health-related quality of life (HRQOL) scores provided by 266 Spanish children with coeliac disease and parents (428) of children with coeliac disease using the specific CDDUX questionnaire.**

| **HRQOL** |  | **N** | **Mean** | **SD** | **Mean differences** | **95% CI** | | **p** |
| --- | --- | --- | --- | --- | --- | --- | --- | --- |
|  |  |  |  |  |  |  |  |  |
| **TOTAL** | **Children** | **266** | **55.48** | **12.72** | **0.66** | **53.9** | **57,0** | **0.499** |
|  | **Parents** | **428** | **54.82** | **12.30** |  | **53.7** | **56,0** |  |
|  |  |  |  |  |  |  |  |  |
| **HAVING COELIAC DISEASE** | **Children** | **266** | **46.52** | **13.08** | **-3.25** | **44.9** | **48,1** | **0.001** |
|  | **Parents** | **428** | **49.77** | **12.60** |  | **48.3** | **51,0** |  |
|  |  |  |  |  |  |  |  |  |
| **COMMUNICATION** | **Children** | **266** | **72.03** | **16.87** | **2.45** | **70,0** | **74,1** | **0.052** |
|  | **Parents** | **428** | **69.58** | **15.65** |  | **68,1** | **71,1** |  |
|  |  |  |  |  |  |  |  |  |
| **DIET** | **Children** | **266** | **51.69** | **16.95** | **1.71** | **49,6** | **53,8** | **0.180** |
|  | **Parents** | **428** | **49.98** | **15.72** |  | **48,5** | **51,5** |  |

***J. Barrio et al. J Pediatr Gastroenterol Nutr. 2016 Apr;62(4):603-8***

**Supplemental digital content. Table 2.**

**Health-related quality of life (HRQOL) scores provided by Spanish children with coeliac disease and parents of children with coeliac disease using the generic KIDSCREEN-52 questionnaire.**

| **DOMAIN** |  | **N** | **MEAN** | **SD** | **95% CI** |  | **P** |
| --- | --- | --- | --- | --- | --- | --- | --- |
| **Social acceptance** | **children** | **255** | **91.92** | **11.82** | **1.19** | **5.15** | **0.001** |
|  | **parents** | **387** | **88.75** | **12.95** | **1.22** | **5.12** |  |
| **Moods and emotions** | **children** | **255** | **83.72** | **13.72** | **-2.82** | **1.16** | **0.427** |
|  | **parents** | **386** | **84.55** | **11.74** | **-2.89** | **1.22** |  |
| **Psychological wellbeing** | **children** | **255** | **70.57** | **9.11** | **0.23** | **3.08** | **0.023** |
|  | **parents** | **387** | **68.91** | **8.92** | **0.22** | **3.09** |  |
| **Physical wellbeing** | **children** | **255** | **65.71** | **8.37** | **0.15** | **2.78** | **0.029** |
|  | **parents** | **387** | **64.25** | **8.26** | **0.14** | **2.78** |  |
| **Self- perception** | **children** | **255** | **58.04** | **10.42** | **-1.50** | **1.49** | **0.998** |
|  | **parents** | **386** | **58.04** | **8.74** | **-1.55** | **1.55** |  |
| **School environment** | **children** | **255** | **58.07** | **5.51** | **0.61** | **2.19** | **0.001** |
|  | **parents** | **387** | **56.67** | **4.64** | **0.58** | **2.22** |  |
| **Parent relations** | **children** | **254** | **50.07** | **5.60** | **0.18** | **1.74** | **0.021** |
|  | **Parents** | **387** | **49.10** | **4.38** | **0.14** | **1.78** |  |
| **Financial resources** | **children** | **254** | **48.06** | **8.79** | **-1.02** | **1.46** | **0.743** |
|  | **Parents** | **386** | **47.84** | **7.09** | **-1.08** | **1.51** |  |
| **Autonomy** | **Children** | **255** | **39.12** | **14.83** | **-1.53** | **2.61** | **0.627** |
|  | **Parents** | **387** | **38.58** | **11.75** | **-1.63** | **2.71** |  |
| **Social support and peers** | **Children** | **255** | **32.18** | **11.91** | **-6.71** | **-2.93** | **0.000** |
|  | **Parents** | **387** | **37.00** | **11.91** | **-6.71** | **-2.93** |  |

**CI = confidence interval**

***J. Barrio, et al. Eur J Pediatr. 2018 ;177(10):1515-1522***
